# Anti‐Modified Protein Antibodies in Rheumatoid Arthritis: Pathogenic, Protective, or Passive Bystander?

**DOI:** 10.1111/imr.70149

**Published:** 2026-07-28

**Authors:** Amber‐Sarai F. L. Stalman, Diane van der Woude

**Affiliations:** ^1^ Department of Rheumatology Leiden University Medical Center Leiden the Netherlands

**Keywords:** ACPA, autoantibodies, pathogenesis, rheumatoid arthritis

## Abstract

The presence of anti‐modified protein antibodies (AMPA) is a hallmark of rheumatoid arthritis (RA). AMPA recognize post‐translationally modified proteins and are cross‐reactive. AMPA include anti‐citrullinated protein antibodies (ACPA), anti‐carbamylated protein antibodies (anti‐CarP), and anti‐acetylated protein antibodies (AAPA). These autoantibodies can be detected years before disease onset and are associated with disease development and progression. Moreover, AMPA have been associated with genetic and environmental risk factors in RA, including human leukocyte antigen (HLA) alleles, smoking, and microbial exposure. Consequently, AMPA have been implicated in RA pathogenesis, yet their exact role remains unclear. ACPA may exert pathogenic, pro‐inflammatory effects through immune complex formation, Fc gamma receptor binding on macrophages, complement activation, and increased neutrophil extracellular trap (NET) formation. Additionally, ACPA have been linked to bone loss and pain induction in mice. However, ACPA may also have protective effects through inhibition of NET release, increased NET clearance, and Fc gamma receptor binding on macrophages. These findings indicate that AMPA may have diverse functions in RA, with both pathogenic and protective effects, whereas some ACPA may not display functional activity. Further studies on AMPA characteristics and mechanisms underlying the pathogenic and protective effects may improve the understanding of the role of AMPA in RA.

## 1 Introduction

Anti‐modified protein antibodies (AMPA) are a hallmark of rheumatoid arthritis (RA) and can be detected years before disease onset. AMPA comprise a group of autoantibodies directed against post‐translationally modified proteins, which include anti‐citrullinated protein antibodies (ACPA), anti‐carbamylated protein antibodies (anti‐CarP), and anti‐acetylated protein antibodies (AAPA) [1, 2, 3]. Because AMPA are associated with disease development and progression, they have also been implicated in RA pathogenesis [4]. RA is a chronic autoimmune disease that affects approximately 0.5%–1% of the population and is more prevalent in women and older individuals [1]. RA is characterized by joint inflammation and often initially affects the small joints of the hands and feet. Various genetic and environmental risk factors associated with the presence of AMPA have been identified. However, the exact role of AMPA in RA remains unclear; for instance, whether they actively promote inflammation, exert protective effects, or reflect ongoing immune dysregulation. This review aims to provide a better understanding of the functional role of AMPA in RA. First, different AMPA and their distinct characteristics are summarized, followed by a description of the genetic and environmental risk factors associated with their presence. Next, the evolution of AMPA throughout the disease course and their associations with clinical outcomes are discussed. Finally, current insights regarding the pathogenic and protective functions of AMPA in RA are reviewed.

## 2 Anti‐Modified Protein Antibodies

ACPA were the first group of autoantibodies to be identified in RA that recognize post‐translational modifications (PTMs) on various proteins. PTMs are the result of physiological processes essential for regulating protein structure and function [5]. As PTMs are inherent to human biology, their presence alone cannot explain the development of AMPA. It remains unclear what mechanisms drive the loss of tolerance toward different PTMs.

### 2.1 Anti‐Citrullinated Protein Antibodies

**FIGURE 1:**
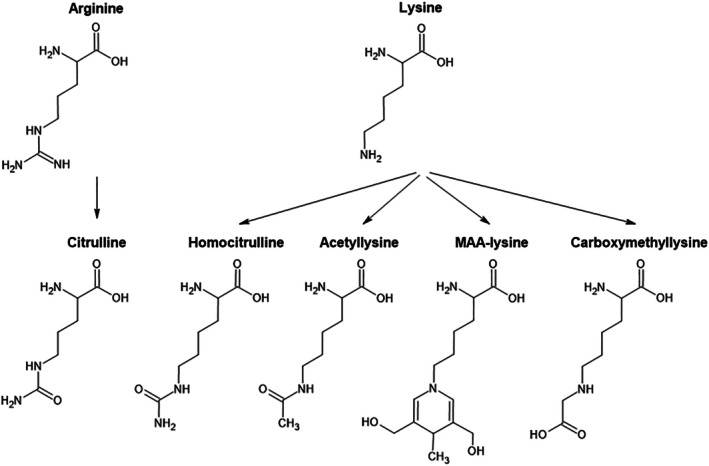
Post‐translational modifications recognized by AMPA in RA. Citrullination is the conversion of arginine into citrulline mediated by peptidyl arginine deiminases (PADs). Carbamylation is a cyanate‐mediated chemical reaction that converts lysine into homocitrulline. Acetylation is an enzyme‐mediated reaction that modifies lysine into acetyllysine. Malondialdehyde‐acetaldehyde adducts (MAA) are formed under conditions of oxidative stress and can react with lysine to form MAA‐lysine. Advanced glycation end‐products (AGEs) are formed through non‐enzymatic glycoxidation, for example: Glycoxidation of lysine residues can generate N^ε^‐carboxymethyllysine (CML).

ACPA are directed against citrullinated proteins, in which arginine residues have been converted into citrulline by peptidyl arginine deiminases (PADs) (Figure 1). ACPA are highly specific for RA and are associated with disease development when found in individuals without RA, and with disease severity once RA has developed [4]. They can be detected up to 14 years before disease onset, with their frequency increasing in the years prior to disease onset [6, 7]. The prevalence of ACPA IgG differs across disease stages, with prevalences of approximately 60% in early RA, which is often defined as a symptom duration of less than 1–2 years, and up to 80% in established RA [8]. In contrast, the frequency of ACPA positivity is much lower in patients with other forms of arthritis or in healthy individuals, with reported prevalences of approximately 6% and 1%, respectively [8].

Most ACPA in serum of RA patients are considered promiscuous [9]. These ACPA can bind to multiple citrullinated epitopes due to their specific recognition of citrulline residues and limited interactions with surrounding residues. Private ACPA, on the other hand, recognize only a selective number of citrullinated epitopes, likely dependent on their interaction with the surrounding amino acid side chains [9]. The overall broad reactivity profiles of ACPA can be attributed to the recognition of consensus binding motifs, in which citrulline is preferentially recognized when adjacent to specific amino acids [10, 11]. In particular, several ACPA clones have been shown to recognize peptides sharing a Cit‐Gly motif. Additionally, binding motifs with serine or threonine in the flanking region of citrulline were also observed. However, not only the binding motif, but also the biochemical properties of amino acids surrounding the binding motif are relevant for recognition, as ACPA clones do not recognize all peptides containing their preferential binding motif [10].

Although numerous citrullinated proteins and peptides recognized by ACPA have been identified, including those derived from filaggrin, vimentin, fibrinogen, and α‐enolase, the antigen responsible for the initial induction of ACPA remains unknown [12, 13]. It also remains unclear whether the ACPA response is initially directed against citrullinated antigens or instead results from cross‐reactivity with other post‐translationally modified antigens.

### 2.2 Anti‐Carbamylated Protein Antibodies

Another class of AMPA are antibodies against carbamylated proteins (anti‐CarP). Carbamylated proteins are generated through a cyanate‐mediated chemical reaction that converts lysine into homocitrulline. Enhanced protein carbamylation can be observed in uremia due to increased cyanate levels [14]. Additionally, elevated cyanate levels have been linked to inflammation, in which myeloperoxidase catalyzes the conversion of thiocyanate into cyanate [14]. Anti‐CarP IgG can be detected in approximately 45% of RA patients [2, 15]. Similar to ACPA, anti‐CarP antibodies are present years before onset of disease and are associated with the development of RA [16, 17]. Notably, anti‐CarP antibodies can also be found in 8%–14% of ACPA‐negative RA patients and are associated with radiological progression in these patients [2, 15]. Anti‐CarP antibodies can be detected in 4%–17% of patients with other forms of arthritis and in 2% of healthy individuals [18]. Anti‐CarP antibodies bind to various carbamylated foreign and self‐proteins [19]. Additionally, many anti‐CarP antibodies are cross‐reactive with other PTMs, while some display specific non‐cross‐reactive binding to carbamylated proteins [20]. The notion that part of the anti‐CarP antibodies do not cross‐react with other PTMs is supported by the presence of anti‐CarP antibodies in a subset of ACPA‐negative RA patients.

### 2.3 Anti‐Acetylated Protein Antibodies

Antibodies against acetylated proteins (AAPA) represent another family of AMPA in RA. Acetylation is a reversible process mediated by acetyltransferases that modify lysine into acetyllysine [3]. AAPA IgG prevalences range from 34% to 60% in early RA patients and up to 69% in established RA patients [21, 22]. AAPA are most frequently detected in ACPA‐positive RA patients but can also be found in ACPA‐negative RA patients, with reported prevalences of 1% in one study and 40% in another study, depending on the assay used [21, 22]. The prevalences of AAPA in healthy controls and patients with other rheumatic diseases are 22% and 7%–31%, respectively [21].

Since bacteria use acetylation as a process to regulate their metabolism, bacterial proteins are extensively acetylated [23]. Acetylated bacterial proteins present at human mucosal surfaces may contribute to the loss of tolerance to self‐proteins in RA. Supporting this hypothesis, a study has demonstrated that chemically acetylated bacterial proteins can be recognized by human AMPA [24]. Furthermore, repeated immunization of mice with these acetylated proteins induced an AMPA response that cross‐reacted with citrullinated, carbamylated, and acetylated fibrinogen. These findings suggest that AMPA elicited by bacterial acetylated proteins can cross‐react with other post‐translationally modified proteins, hence representing a potential starting point for the AMPA response.

### 2.4 Anti‐MAA and Anti‐AGE Antibodies

In addition to the AMPA discussed thus far, autoantibodies directed against other PTMs have been described in patients with RA and other forms of arthritis, including autoantibodies against malondialdehyde‐acetaldehyde adducts (MAA) and advanced glycation end‐products (AGE) [25, 26, 27]. MAA are formed as a result of oxidative stress, whereas AGE are formed through non‐enzymatic glycation and glycoxidation reactions. Anti‐MAA and anti‐AGE antibodies are present in approximately 46% and 45% of early RA patients, respectively [26]. Notably, anti‐MAA and anti‐AGE antibodies can also be detected in 29% and 34% of ACPA‐negative RA patients, respectively [26]. In this subgroup, both anti‐MAA and anti‐AGE are associated with HLA‐DRB1*03. However, a similar association with HLA‐DRB1*03 was also observed in patients with other forms of arthritis. Along the same lines, anti‐MAA antibodies have been described to be associated with inflammatory markers, such as CRP, in both patients with RA and other forms of arthritis. Furthermore, anti‐MAA and anti‐AGE antibodies are associated with radiographic joint damage in RA patients. Nevertheless, anti‐MAA and anti‐AGE antibodies can also be detected in 30% and 33% of patients with other forms of arthritis, respectively, indicating a lack of specificity for RA [26]. This low specificity, and the fact that anti‐MAA and anti‐AGE antibodies less frequently co‐occur with ACPA in comparison to anti‐CarP antibodies and AAPA, suggest that anti‐MAA and anti‐AGE represent autoantibody responses which are distinct from the other AMPA responses described above. Accordingly, anti‐MAA and anti‐AGE antibodies may arise through different mechanisms and exhibit different roles in RA pathogenesis than other AMPA. Because of these differences, and because these autoantibodies are less specific for RA, this review will further focus on the characteristics and functional roles of ACPA, anti‐CarP antibodies, and AAPA.

## 3 AMPA Characteristics

The immunological features of AMPA have been extensively studied, with T cell dependent immune responses frequently implicated. Since antibody characteristics reflect the immunological processes that shape the immune response, including affinity maturation, T cell involvement, and cytokine signaling, studying AMPA characteristics may provide valuable insights into mechanisms driving AMPA development. This section discusses the key features of AMPA.

### 3.1 AMPA IgM

Although AMPA responses are often determined based on methods that measure IgG, other isotypes, including IgM and IgA, can also be detected. IgM is the first isotype produced during an antibody response. Upon receiving T cell help, B cells undergo avidity maturation and class‐switching resulting in the production of other isotypes. Studies investigating patterns of ACPA isotypes in pre‐disease individuals have demonstrated that levels of all isotypes are elevated, with the greatest increase directly prior to disease onset [28, 29]. One study reported that ACPA IgG positivity could be detected earlier than ACPA IgM and ACPA IgA positivity, with median times before disease onset of 1.9, 1.6, and 1.4 years, respectively [28]. Another study reported that ACPA IgG and ACPA IgA positivity preceded that of ACPA IgM [29]. Overall, these findings indicate that ACPA responses develop years before disease onset, with a typical isotype progression in which IgG is detected first, followed by IgA and IgM. The absence of a detectable early phase in which ACPA IgM precedes ACPA IgG in pre‐disease samples most likely indicates a transient IgM response that rapidly undergoes isotype switching. Consequently, the initial phase in which ACPA IgM is present may be short‐lived and hence not detected. The later detection of IgM may reflect ongoing immune activation with continuous recruitment and activation of B cells in response to persistent antigen exposure.

The presence of ACPA IgM and ACPA IgA is largely restricted to ACPA IgG‐positive patients, with each of these isotypes being detected in approximately 61% and 62% of RA patients, respectively [30]. Anti‐CarP isotypes, on the other hand, could be detected in both ACPA‐positive and ACPA‐negative RA patients [31]. Anti‐CarP IgM and anti‐CarP IgA are present in approximately 16% and 45% of all RA patients, respectively [31]. In ACPA‐negative RA patients, the prevalence of anti‐CarP IgM is approximately 5% and that of anti‐CarP IgA 24% [31]. Anti‐CarP IgM and anti‐CarP IgA can also be found in some anti‐CarP IgG‐negative patients. Similar to anti‐CarP, AAPA isotypes could be detected in both ACPA‐positive and ACPA‐negative RA patients, even though AAPA IgG is largely restricted to ACPA IgG‐positive patients [21, 22]. Furthermore, AAPA IgM and AAPA IgA can also be detected in 8% and 2% of AAPA IgG‐negative patients, respectively [22]. Notably, high levels of AAPA IgM cannot only be detected in RA patients, but also in healthy individuals [32]. This suggests that AAPA IgM may be part of the general immune repertoire and could represent a potential starting point for the induction of AMPA in RA [32].

### 3.2 AMPA IgA

As mucosal immune responses have been hypothesized to play a role in the pathophysiology of RA, studying characteristics of IgA has been of particular interest. IgA comprises two subclasses, IgA1 and IgA2, which are structurally different in their hinge region and glycosylation patterns [33, 34]. In serum, approximately 90% of IgA is IgA1 and 10% is IgA2 [33]. At mucosal sites, the proportions of these subclasses can vary dependent on the location. IgA2 was shown to have pro‐inflammatory effects on macrophages and neutrophils, while this was not observed for IgA1 [34]. Therefore, the distinction between IgA1 and IgA2 subclasses might be relevant when studying associations with disease activity in RA. Indeed, the distribution of total IgA1 and total IgA2 was slightly shifted toward IgA2 in serum of RA patients [34]. This shift toward IgA2 was more pronounced when studying ACPA IgA1 and IgA2. Higher proportions of ACPA IgA1 were linked to lower disease activity, whereas higher proportions of ACPA IgA2 were associated with increased disease activity. In contrast, another study reported elevated levels of both total IgA1 and IgA2 in RA patients compared to healthy controls but found no relative increase of IgA2 [35]. In this study, total IgA2 was not associated with CRP, but it was linked to smoking. This suggests that mucosal immune responses may be involved, as smoking can promote inflammation at mucosal sites. Thus, it remains to be elucidated whether there is a substantial increase in IgA2 in RA and whether this plays a role in the pathophysiology of this disease.

Another feature of IgA is that it can be present in monomeric or dimeric form, the latter consisting of two IgA molecules with a J‐chain. IgA is mainly present in monomeric form in serum, whereas at mucosal sites IgA is mainly produced in dimeric form. Intriguingly, a higher percentage of dimeric IgA was found for AMPA IgA compared to total IgA in serum (65% vs. 20%, respectively) [36]. Similar results were found for other rheumatic autoimmune diseases, including systemic lupus erythematosus (SLE) and anti‐neutrophilic cytoplasmic antibodies (ANCA)‐associated vasculitis (AAV) [36]. Further investigations into the underlying mechanism of this increased dimerization revealed that a higher percentage of dimeric IgA is also found shortly after intramuscular SARS‐CoV‐2 vaccination in healthy individuals [36]. These findings suggest that the formation of dimeric IgA could be associated with recent immune activation. Taken together, increased dimerization of AMPA IgA may point to a mucosal origin, whereas it could also reflect ongoing immune activation in RA, the latter being supported by the continuous presence of AMPA IgM.

### 3.3 Cross‐Reactivity

A defining feature of AMPA is their extensive cross‐reactivity with multiple PTMs [37]. This cross‐reactivity was not only observed for AMPA IgG, but also for AMPA IgM [38]. When reverting AMPA IgM back to germline sequences, cross‐reactivity with multiple PTMs could still be observed, suggesting that this characteristic is not acquired due to somatic hypermutation [38]. Additionally, AMPA cross‐reactivity can already be observed in pre‐RA individuals, indicating that cross‐reactivity is an intrinsic feature of AMPA [39]. Consistent with human data, mechanistic studies in mice showed that immunization with either carbamylated or acetylated ovalbumin induced an AMPA response that was cross‐reactive toward both carbamylated and acetylated proteins [40, 41]. Notably, booster immunization with antigens carrying a different PTM than the initial immunization increased reactivity toward the booster PTM [41]. This suggests that AMPA reactivity toward one PTM may be skewed upon exposure to another PTM, leading to increased reactivity toward that other PTM. Importantly, a recent study has shown that detection of AMPA cross‐reactivity can be affected by the antigenic backbones used in the assay [42]. Specifically, assays using a cyclic modified peptide backbone identified a larger proportion of patients with reactivity to multiple PTMs than assays using a protein backbone. Therefore, when investigating AMPA cross‐reactivity profiles, the antigenic backbone used in the assay should be considered. In conclusion, studies regarding cross‐reactivity of AMPA have led to the insight that the AMPA response may be elicited against one PTM, and shifted toward other PTMs upon exposure to these PTMs. Thus, the origin of the AMPA response may potentially be a break in tolerance toward a single PTM, which could eventually be the starting point for the broader AMPA response.

### 3.4 Altered Fc Glycosylation

AMPA in RA are characterized by altered glycosylation patterns. The Fc region of human IgG contains two conserved N‐linked glycosylation sites located at Asn297 on each heavy chain [43]. Fc glycosylation is essential for maintaining structural stability and mediating effector functions, including complement activation and antibody‐dependent cellular cytotoxicity through Fc gamma receptor (FcγR) binding. Consequently, alterations in the Fc glycan profile of ACPA IgG may impact Fc‐mediated effector mechanisms that could contribute to RA pathogenesis. In serum of RA patients, reduced galactosylation of both total IgG and ACPA IgG has been observed prior to disease onset [44, 45, 46]. This reduction was observed 3 months before diagnosis and was not observed in patients with undifferentiated arthritis [45]. Reduced galactosylation of Fc glycans was also observed for other rheumatic diseases, including SLE and AAV, as well as for other inflammatory diseases [43]. Decreased levels of galactosylation correlated with disease activity and inflammatory markers in RA [44, 45, 46]. Interestingly, the improvement in disease activity that is observed during pregnancy in RA patients was also associated with changes in ACPA IgG galactosylation [47]. Thus, although altered Fc galactosylation is not specific for RA, it may still serve as a biomarker in RA because of its association with disease activity and systemic inflammation.

### 3.5 Extensive V‐Domain Glycosylation

A striking feature of ACPA IgG is the presence of extensive N‐glycosylation within their variable (V) domains. This feature was initially identified by the observation that the majority of ACPA IgG had a higher molecular weight in comparison to other IgG molecules [48]. This difference in molecular weight could be explained by the presence of N‐glycans in the V‐domains in over 90% of ACPA IgG compared with only 15%–20% of other IgG molecules [49]. Additionally, V‐domain glycosylation levels of 51% are detected in anti‐CarP IgG [50]. Structural analyses with Liquid Chromatography‐Mass Spectrometry (LC–MS) revealed that these V‐domain glycans were highly sialylated with a high frequency of di‐galactosylation [49]. In synovial fluid, V‐domain glycosylation levels of ACPA IgG were even higher than in plasma [49]. This might indicate an increased inflammatory potential of ACPA in synovial fluid, which was also suggested based on Fc glycosylation patterns of ACPA in synovial fluid [46]. Notably, no molecular size shift was observed for ACPA IgM, indicating that this isotype is not characterized by extensive V‐domain glycosylation [51]. N‐glycosylation requires the presence of the consensus sequence N‐X‐S/T, where X represents any amino acid except proline. Consistent with the high prevalence of glycosylation described above, these N‐glycosylation sites are present at high frequencies within the V‐domains of ACPA IgG [52]. B cell receptor (BCR) sequencing has revealed that these N‐glycosylation sites are not germline encoded, but introduced by somatic hypermutation. This implies the involvement of T cell dependent immune responses.

Production of monoclonal antibodies with and without V‐domain glycans enabled further investigation into the functional consequences of V‐domain glycosylation on ACPA IgG. Crystal structures of ACPA IgG showed that V‐domain glycans were located in close proximity to the antigen‐binding pocket, suggesting that they could affect the binding of the antigen [53]. Indeed, ACPA IgG with V‐domain glycans showed reduced binding to different citrullinated proteins and peptides compared to ACPA IgG without glycans [48, 53]. The binding to several citrullinated antigens improved when sialic acids were cleaved off the V‐domain glycans, while the absence of all V‐domain glycans resulted in even higher binding capacities [53].

Furthermore, the functional consequences of V‐domain glycans present on BCRs were investigated in a Ramos B cell line that expressed ACPA BCRs with or without V‐domain glycans [53]. B cells expressing BCRs with V‐domain glycans showed reduced antigen binding compared to B cells expressing BCRs without V‐domain glycans. Intriguingly, additional analyses using Ramos B cell lines showed that BCRs with V‐domain glycans are nonetheless more potently activated by citrullinated peptide tetramers, as measured by higher calcium flux and phosphorylation of spleen tyrosine kinase (pSyk), a key molecule in B cell signaling [53]. Furthermore, BCRs with V‐domain glycans stayed longer on the B cell surface upon antigen binding compared to BCRs without V‐domain glycans. Thus, there appear to be several mechanisms with opposing actions at play: V‐domain glycans negatively affect binding of both secreted antibodies and BCRs to their antigen, while they positively affect BCR activation and downregulation, meaning that they lead to more potent B cell activation. These data give insights in potential mechanisms by which increased V‐domain glycosylation on ACPA IgG results in selection advantages to autoreactive B cells.

## 4 Genetic and Environmental Risk Factors

The development of RA is likely driven by a complex interplay between genetic susceptibility and environmental exposures. This section summarizes the key genetic and environmental risk factors to better understand how they can contribute to the initiation and/or progression of disease.

### 4.1 HLA

The most important genetic risk factor in RA is the human leukocyte antigen (HLA) region. HLA‐DRB1 alleles encoding a specific amino‐acid sequence motif at position 70–74, also known as the shared epitope (SE), are significantly associated with ACPA‐positive RA (OR = 4.75, 95% CI 3.54–6.38) [15, 22, 54]. When examining associations of ACPA fine specificities with HLA‐SE alleles, associations are observed for some, but not all fine specificities [13]. Notably, the association with HLA‐SE alleles is shown to be restricted to ACPA specificities recognizing non‐glycine citrulline motifs [55]. Furthermore, HLA‐SE alleles are associated with elevated levels of ACPA V‐domain glycosylation before disease onset [56]. However, this association is no longer observed in patients with established RA, likely due to the high levels of ACPA glycosylation already present at that stage. In contrast to ACPA, anti‐CarP antibodies were not associated with HLA‐SE alleles [15]. Instead, anti‐CarP antibodies were associated with HLA‐DRB1*03 alleles in ACPA‐negative RA [57]. However, another study showed that anti‐CarP antibodies were associated with HLA‐B*08 and that the previously found association with HLA‐DRB1*03 could be explained by linkage disequilibrium [58]. Although an association between AAPA and HLA‐SE was initially found, this association was no longer present after adjusting for ACPA positivity [22, 27].

A leading hypothesis for the association between HLA‐SE alleles with ACPA is that the SE motif affects peptide presentation to CD4+ T cells [59, 60]. The SE motif is positioned in the peptide‐binding groove of HLA class II molecules and specifically influences the structure of the P4 pocket. In HLA‐DRB1*04:01 transgenic mice, it was shown that citrullinated peptides were bound with higher affinity than their corresponding non‐citrullinated peptides [59]. Crystal structures of HLA‐DRB1‐antigen complexes further revealed that the positively charged P4 pockets of HLA‐DRB1*04:01 and HLA‐DRB1*04:04 preferentially bind citrulline over arginine residues [60]. In contrast, HLA‐DRB1*04:02, which lacks the SE, has a negatively charged P4 pocket that accommodates both citrulline and arginine residues [60]. The preferential binding of citrullinated peptides by HLA‐SE molecules could consequently lead to enhanced peptide presentation to CD4+ T cells.

In contrast to associations described for HLA‐SE, protective effects of HLA alleles in association with ACPA have also been found. HLA‐DRB1*13, particularly the HLA‐DRB1*13:01 allele, has been associated with protection from ACPA‐positive RA (OR = 0.54, 95% CI 0.38–0.77) [61, 62]. A hypothesis explaining the protective effects of HLA‐DRB1*13 involves the DERAA sequence [63]. This sequence cannot only be found in HLA‐DRB1*13, but also in numerous bacteria and viruses, as well as in the autoantigen citrullinated vinculin. The hypothesis proposes that T cells initially directed against microbial DERAA epitopes may cross‐react with the DERAA epitope of citrullinated vinculin, thereby priming these T cells to provide help to ACPA B cells [63]. However, in carriers of protective HLA alleles, these T cells would undergo negative selection in the thymus due to presentation of the DERAA sequence derived from HLA‐DRB1*13. Consequently, DERAA‐specific T cells would be eliminated or tolerized in HLA DRB1*13:01‐positive individuals, preventing them from providing T cell help to B cells targeting citrullinated vinculin. Overall, the association with HLA risk alleles suggests a T cell dependent immune response contributing to AMPA maturation, consistent with the observed extensive somatic hypermutation and class switch recombination of AMPA.

### 4.2 PTPN22

Besides HLA risk alleles, another relevant genetic risk factor is the protein tyrosine phosphatase gene N22 (PTPN22), in which the missense single‐nucleotide polymorphism C1858T (R620W) has been associated with ACPA‐positive RA [64]. In contrast, this association was not found for anti‐CarP antibodies [15]. The presence of the PTPN22 missense mutation could interfere with BCR signaling, resulting in impaired central B cell tolerance. Consequently, presentation of autoantigens to autoreactive B cells may not result in the deletion of these B cells, which could facilitate autoantibody production [65, 66].

In conclusion, of the genetic risk factors currently identified in RA, the vast majority are associated with seropositive disease. This suggests that seropositive and seronegative RA may have distinct pathophysiological mechanisms. Notably, many genetic risk factors are linked to the adaptive immune system, suggesting a prominent role in seropositive disease.

### 4.3 Smoking

Smoking is a strong environmental risk factor for ACPA‐positive RA. Smoking was not solely associated with the presence of ACPA, but particularly with the presence of two (OR = 1.32, 95% CI 1.04–1.68) or three autoantibodies (OR = 2.05, 95% CI 1.53–2.73) (ACPA, rheumatoid factor (RF) and anti‐CarP antibodies) [67]. No association with smoking was found for anti‐CarP antibodies [15]. Further analyses of isotypes showed that, after correction for AMPA IgG, the association with smoking was predominantly present for ACPA IgA (OR = 1.89, 95% CI 1.14–3.12) and AAPA IgA (OR = 2.30, 95% CI 1.35–3.94) [22]. Previous studies have described a gene–environment interaction between HLA‐SE and smoking for ACPA in RA, with combined odds ratios that are higher than the sum of the individual odds ratios [68, 69]. Interestingly, the gene–environment interaction between HLA‐SE and ACPA was observed in patients who were double positive for ACPA IgA and ACPA IgG, but not for patients who were positive for ACPA IgG only [22]. As smoking acts as a mucosal stimulus in the lungs, an association with IgA is not unexpected, given that IgA is the predominant antibody present at mucosal sites. Therefore, the link between smoking and AMPA IgA supports the hypothesis that mucosal immune responses may play a role in the development of RA.

A potential explanation for this association is that smoking could increase PAD expression in the lung and therefore lead to increased amounts of citrullinated proteins. An increase in citrullinated proteins and PAD2 expression was observed in the lungs of healthy smokers compared to those of non‐smokers [67, 70]. Additionally, associations between other inhalable exposures, such as silica dust, air pollution, and occupational inhalants and development of RA have been described [71]. The detection of autoantibodies in sputum and bronchoalveolar lavage fluid further suggests that these autoantibodies can be locally produced, highlighting the potential role of mucosal sites in the origin of AMPA [72, 73].

### 4.4 Microbial Exposure at Mucosal Surfaces

The mucosal origin hypothesis suggests that immune responses at mucosal surfaces play a key role in the development of RA [74]. Disruption of the mucosal epithelial barrier, for example due to inflammatory processes or microbial dysbiosis, could facilitate translocation of post‐translationally modified microbial products, triggering immune activation. This could promote T cell help to B cells, leading to their activation. Activated B cells may subsequently migrate to systemic sites or joints, where they can lead or contribute to inflammation.

Besides the respiratory mucosa, the oral mucosa is another potential site that could contribute to AMPA production, since AMPA can be detected in saliva of RA patients [75, 76]. ACPA IgA levels in saliva correlated with levels in serum and are associated with higher disease activity and inflammatory markers in RA patients [77]. Additionally, chronic periodontitis has long been associated with RA [78]. Oral pathogens implicated in RA include *Porphyromonas gingivalis* ( *P. gingivalis* ) and *Aggregatibacter actinomycetemcomitans*. Antibodies against *P. gingivalis* were elevated in RA patients compared to control patients, which was mainly observed in ACPA‐positive RA patients [79, 80]. These antibody levels were already found to be increased years before the onset of disease, and did not associate with smoking or HLA‐SE alleles [80]. *P. gingivalis* expresses a bacterial peptidyl arginine deiminase (PPAD) capable of citrullinating bacterial and host proteins [81]. This may promote the loss of immune tolerance toward citrullinated autoantigens through molecular mimicry. Indeed, citrullinated α‐enolase peptide derived from humans and *P. gingivalis* showed high sequence similarity. After citrullination of *P. gingivalis* enolase with rabbit skeletal PAD in vitro, antibodies from RA patients were found to cross‐react with both citrullinated human and bacterial α‐enolase [82]. However, cross‐reactive binding of ACPA to autocitrullinated *P. gingivalis* proteins (citrullination by PPAD) could not be shown in early RA patients [83]. *Aggregatibacter actinomycetemcomitans*, on the other hand, can induce hypercitrullination of neutrophils via leukotoxin A, thereby dysregulating citrullination and increasing the presence of citrullinated autoantigens [84].

Moreover, microbial dysbiosis in the gut has also been described as a risk factor for RA. For example, enrichment of *Segatella copri* (formerly *Prevotella copri* ) was found in stool samples of at‐risk individuals and patients with new‐onset RA [85, 86]. Another gut commensal described in association with RA is *Subdoligranulum didolesgii*. Antibodies against this bacterium have been detected in serum of individuals at risk of RA and have been shown to promote autoantibody production and joint inflammation in mice [87]. When evaluating whether there is any evidence for local production of AMPA in the human intestine, AMPA could not be detected in feces or ileal washes of RA patients [76]. This suggests that the lower intestinal mucosa may be a less prominent site of local autoantibody production, raising the question of whether exposure to post‐translationally modified proteins at this site indeed contributes to AMPA induction. Alternatively, different mechanisms within the gut or other mucosal sites may be more relevant in the AMPA response.

### 4.5 Timing of Risk Factors in Relation to Disease Progression

Recent meta‐analyses have shown that smoking and HLA‐SE exert their effects at different disease stages [88]. Smoking is primarily involved in the development of ACPA (OR = 1.37, 95% CI 1.15–1.63), while no such association was observed for HLA‐SE [88]. In contrast, HLA‐SE, but not smoking, is implicated in the progression of (clinically suspect) arthralgia to clinical arthritis (HR = 1.52, 95% CI 1.08–2.15) [88]. In turn, both smoking and HLA‐SE were associated with the development of symptoms in ACPA‐positive individuals, while no such associations were found for RF, anti‐CarP, and AAPA positivity [88]. These findings suggest that initial ACPA development may involve HLA‐SE‐independent T cell responses, whereas HLA‐SE‐dependent T cell responses may become more prominent during symptom onset and progression to RA.

In conclusion, several genetic and environmental risk factors have been linked to the development of RA, with associations predominantly observed in seropositive disease. These risk factors appear to influence disease progression at different disease stages, with smoking being relevant to AMPA development and early symptoms, and HLA‐SE being associated with early symptoms and progression to RA.

## 5 Evolution of the AMPA Response

**FIGURE 2:**
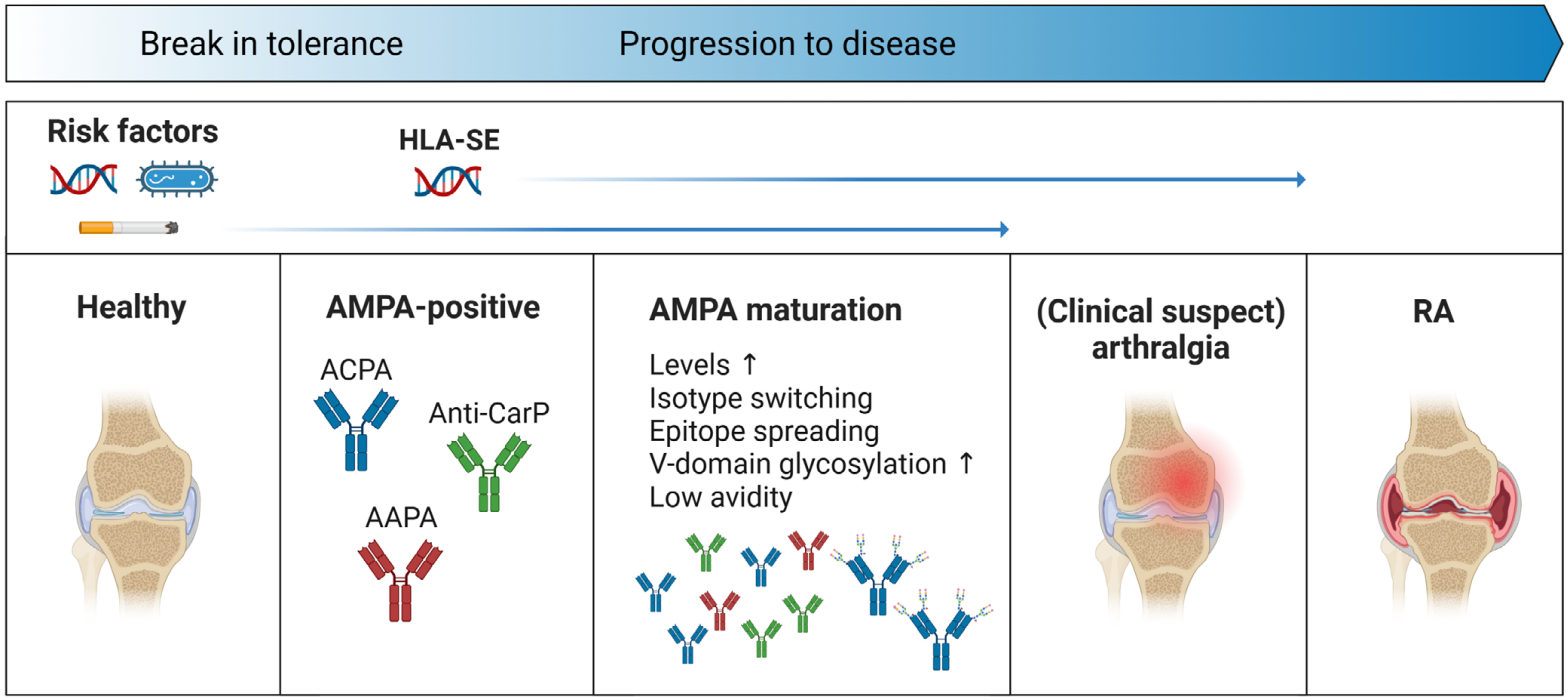
Different disease stages in the development of seropositive RA. The development of seropositive RA is often described as a multistep process, starting with a break in tolerance toward post‐translationally modified proteins years before the onset of disease. This leads to systemic autoimmunity characterized by the presence of autoantibodies. Maturation of the AMPA response may subsequently drive disease progression, resulting in the development of (clinical suspect) arthralgia and clinical RA. Genetics, smoking, and microbial dysbiosis are relevant risk factors in RA development. Smoking is primarily involved in the development of AMPA and their maturation. In contrast, HLA‐SE alleles have been shown to be associated with the development of symptoms in autoantibody‐positive individuals, but not the initial development of autoantibodies. HLA alleles other than HLA‐SE may thus contribute to the initial break in tolerance. Microbial dysbiosis may lead to a break in tolerance, while a role at other stages of disease development could also be possible. Created in BioRender. Stalman, A. (2026). https://BioRender.com/4k8m12y.

The development of seropositive RA is hypothesized to be a multistep process in which multiple “hits” are required for disease onset. It is thought to begin with a break in immune tolerance to post‐translationally modified proteins, leading to development of systemic autoimmunity. The phase preceding RA (pre‐RA) comprises an asymptomatic stage and a symptomatic stage during which patients experience symptoms such as arthralgia, which is associated with an increased risk of progression to RA [89]. Progression from pre‐RA to clinical RA is thought to require additional, yet unknown, “hits” that result in maturation of the AMPA response. Understanding how the AMPA response develops may provide insights into its role in RA pathogenesis (Figure 2).

### 5.1 Autoreactive B Cells

AMPA are produced by autoreactive B cells that have differentiated into plasmablasts or plasma cells. In a healthy situation, multiple B cell tolerance checkpoints ensure that tolerance toward autoantigens is maintained and autoreactive B cells are eliminated. In the bone marrow, 55%–75% of immature B cells display self‐reactivity [90]. Central B cell tolerance ensures the elimination of these autoreactive B cells via negative selection. Immature B cells that recognize autoantigens undergo receptor editing, are subjected to clonal deletion, or become anergic [91]. However, central B cell tolerance is incomplete, allowing potentially autoreactive B cells to migrate to the periphery. Peripheral B cell tolerance mechanisms further regulate and limit their activation. Defects in central or peripheral B cell tolerance checkpoints may result in the presence of AMPA‐expressing B cells. These B cells are likely activated through T cell dependent germinal center responses, since the AMPA response is characterized by extensive somatic hypermutation and class‐switching, and strong associations with HLA [92].

The development of a method using tetramers of citrullinated peptides to stain B cells antigen‐specifically has enabled the identification and isolation of AMPA‐expressing B cells, which have been detected in peripheral blood and synovial fluid of RA patients [92, 93, 94]. AMPA‐expressing B cells have been shown to display phenotypic markers of highly activated and proliferative memory B cells and plasmablasts (such as CD80, CD86, and Ki‐67) [92, 93, 94]. In addition, ACPA memory B cells also have enhanced phosphorylation of spleen tyrosine kinase (SYK), Bruton's tyrosine kinase (BTK), serine/threonine‐specific protein kinase AKT (AKT), and ribosomal protein S6, which is indicative of active BCR signaling [95]. During the disease course, the activated phenotype of ACPA memory B cells is most prominent at the onset of RA, while in arthralgia patients this phenotype is less pronounced [94]. Interestingly, this activated phenotype is still present in patients who are in clinical remission, indicating that these patients do not reach immunological remission [94]. These observations highlight the continuous activation of AMPA‐expressing B cells which likely contributes to the maturation and diversification of the AMPA response.

### 5.2 Progression From Preclinical to Clinical RA

AMPA can be detected years before the onset of clinical symptoms and their presence is associated with progression to disease [2, 4, 6, 16]. However, not all individuals who are AMPA positive develop clinical RA. In a large Dutch population‐based cohort, only 22% of ACPA‐positive individuals were reported to have RA [96]. Additionally, in a study with 45 ACPA‐positive at risk individuals, only 36% progressed to clinical RA [97]. This indicates that solely the presence of AMPA is not sufficient to drive RA pathogenesis.

Biomarkers for progression toward RA include a rise in AMPA positivity and serum levels several years before diagnosis of RA (shown for ACPA and anti‐CarP antibodies), and increased isotype usage [6, 16, 28, 29]. Epitope spreading of the ACPA response can be observed prior to disease onset, resulting in increased reactivity against citrullinated peptides in RA patients compared to pre‐RA patients [98, 99]. In addition, extensive ACPA IgG V‐domain glycosylation has been shown to be associated with progression toward disease. V‐domain glycosylation could already be detected up to 15 years before disease with an increase of levels of V‐domain glycosylation before disease onset [100].

However, there are also studies that did not observe a rise in AMPA levels more closely to disease onset. In an analysis of 13 ACPA‐positive at risk individuals who progressed toward RA, no increase in ACPA levels before disease onset could be observed [97]. Furthermore, in patients with clinically suspect arthralgia (CSA), it was shown that neither levels of ACPA, anti‐CarP, and AAPA, nor the number of isotypes increased as the disease progressed to inflammatory arthritis or RA [89]. Similar findings were reported for ACPA isotypes in patients with undifferentiated arthritis (UA) who progressed to RA [101]. In line with these observations, ACPA reactivities showed no differences between arthralgia patients who developed clinical arthritis and those who did not [102]. In another study, however, patients with UA who developed RA showed increased recognition of citrullinated antigens compared to those who did not develop RA [99]. Longitudinal analysis revealed that ACPA fine specificities did not expand during progression from UA to RA. A potential explanation for these observations is that maturation of the AMPA response may already have occurred before the symptomatic stage, whether this is arthralgia or UA, and consequently does not expand further upon progression to RA.

### 5.3 Limited Avidity Maturation

Despite extensive isotype switching, epitope spreading, and somatic hypermutation, ACPA have low avidity for their antigens compared with antibodies directed against recall antigens such as tetanus toxoid [103]. In addition, anti‐CarP avidity was shown to be even lower than that of ACPA [104]. While an increase in ACPA avidity occurs before disease onset, this was not observed for anti‐CarP antibodies [104, 105]. ACPA avidity was lower in ACPA‐positive healthy individuals than in patients with arthralgia or UA [105]. In symptomatic patients, ACPA avidity varied between patients, with most patients demonstrating limited avidity maturation. No differences in ACPA avidity were observed in patients with arthralgia or UA who progressed to RA compared to those who did not. In addition, no changes in ACPA avidity were observed during follow‐up after onset of RA. Hence, ACPA avidity cannot be regarded as a prognostic biomarker [103, 105]. Overall, the lower avidity of AMPA suggests that avidity maturation is a process that is disconnected from extensive isotype switching, while these processes typically co‐occur. One hypothesis is that this is related to the widespread presence of citrullinated antigens [103, 105]. This high availability may reduce competition between B cells for antigens within germinal centers, thereby limiting avidity maturation. Alternatively, other potential explanations involve features acquired during AMPA maturation, such as V‐domain glycosylation, which may provide selective advantages for B cells without the need for avidity maturation [49].

In summary, maturation of the AMPA response encompasses extensive isotype switching, epitope spreading, increased V‐domain glycosylation, and limited avidity maturation. Several studies suggest that maturation of the AMPA response occurs primarily before the onset of symptoms, as no significant changes are observed in patients with CSA or UA who progress to RA. Overall, an important question remains whether features acquired during AMPA maturation actively drive disease progression and may therefore represent a potential target for therapeutic interventions to halt this process, or whether they instead reflect consequences of the underlying disease process.

## 6 AMPA and Clinical Outcomes

Associations between AMPA and clinical outcomes have been extensively studied to better understand their potential role as biomarkers in RA. Clinical outcomes include disease activity, early treatment response, and long‐term disease outcomes, such as radiographic progression and sustained DMARD‐free remission (SDFR). The following section outlines the associations between AMPA and these clinical outcomes.

### 6.1 Disease Activity at Clinical Presentation

Perhaps surprisingly, considering the differences in risk factors described above, no clear distinction can be observed between the clinical presentation of ACPA‐positive and ACPA‐negative RA patients at disease onset [4]. Early symptoms such as morning stiffness and the number and distribution of tender and swollen joints are similar between the two patient groups, as are CRP levels. In contrast, the presence of anti‐CarP antibodies has been associated with higher disease activity and increased inflammatory markers (CRP or ESR) at baseline, irrespective of ACPA or RF status [106, 107]. Moreover, patients with a higher number of autoantibodies had a longer symptom duration at the time of diagnosis and higher ESR levels compared to those with fewer autoantibodies [108]. Overall, these findings suggest that some AMPA responses, and in particular the overall breadth of the AMPA response, are associated with clinical phenotype at baseline.

### 6.2 Treatment Response

Treatment response can be assessed using different measures, including the disease activity score based on 28 joint counts (DAS28) or 44 joint counts (DAS44). Other measures for disease activity include the Simplified Disease Activity Index (SDAI) and Clinical Disease Activity Index (CDAI). In addition, treatment response can be defined using EULAR response criteria based on DAS28 changes or ACR response criteria based on the percentage of improvement in joint counts and additional disease activity measures.

No differences were observed in early treatment response to methotrexate between seropositive and seronegative RA patients [109]. The proportion of patients that achieved DAS‐remission was similar for both groups. Moreover, there were no differences in physical function, as measured by the Health Assessment Questionnaire (HAQ). Consistent with these findings, two DAS‐steered treatment strategy studies reported no differences in DAS and functional ability between ACPA‐positive and ACPA‐negative RA patients [110, 111]. However, among seropositive RA patients, a broader baseline autoantibody profile, including ACPA, anti‐CarP, AAPA, and RF isotypes, was associated with a slightly better early treatment response, as measured by a greater DAS change after 4 months of treatment with methotrexate [112]. The observation that treatment response is associated with the breadth of the autoantibody profile rather than with any single AMPA is consistent with previous findings on disease activity at first clinical presentation.

Furthermore, the association of a broad autoantibody profile with a better treatment response was also observed in ACPA‐positive patients with arthralgia. A phase IIb study showed that treatment with abatacept in these patients may reduce the progression to clinical arthritis [113]. While a broader autoantibody profile and high ACPA IgG levels were associated with a higher risk of progression to RA, they were also associated with a better treatment response to abatacept compared to placebo [114].

In patients with established RA, several studies have investigated possible associations between autoantibody status and treatment responses to biological disease‐modifying anti‐rheumatic drugs (bDMARDs). A registry‐based study reported a comparable treatment response to abatacept after 1 year for ACPA‐positive and ACPA‐negative RA patients [115]. Nonetheless, abatacept discontinuation rates were lower in seropositive patients. Furthermore, a meta‐analysis investigating the response to treatment with rituximab reported that seropositive RA patients had a greater DAS reduction than seronegative RA patients [116]. Notably, this effect was primarily observed in patients who had previously failed on TNF inhibitors. Another meta‐analysis assessing the overall treatment response to different bDMARDs showed no differences between seropositive and seronegative RA patients [117]. Only in patients who had not responded to TNF inhibitors did treatment response to bDMARDs seem to be slightly better in seropositive RA, although the number of studies was small. Furthermore, the authors of this last study suggested that a possible explanation for this difference could also be misdiagnosis in seronegative RA patients. If these patients do not respond to previous treatments, including TNF inhibitors, one can wonder whether their disease was amenable to anti‐inflammatory treatment at all, or whether there may be very heterogeneous underlying pathophysiological mechanisms at play in this subgroup. Taken together, the treatment response to bDMARDs appears largely comparable between seropositive and seronegative RA patients. Only in patients who had previously not responded to TNF inhibitors, seropositivity may be associated with a better treatment response to bDMARDs.

### 6.3 Long‐Term Disease Outcomes

ACPA have been associated with a more severe disease course. Long‐term disease outcomes include radiographic progression, DMARD‐free remission (DFR), and sustained DMARD‐free remission (SDFR). Radiographic progression is a measure of joint damage and can be assessed using scoring methods such as the Sharp‐van der Heijde Score (SHS) or the Larsen score. DFR refers to remission after discontinuation of DMARDs, and SDFR is defined as DMARD‐free remission over a prolonged period of time.

ACPA‐positive patients had higher disease activity and more severe radiographic progression than ACPA‐negative patients at follow‐up [4, 111, 118]. However, when examining ACPA characteristics, distinct ACPA fine specificities did not show associations with disease activity or radiographic progression [13, 119]. Anti‐CarP antibodies have been linked to radiographic progression in RA, particularly in ACPA‐negative RA patients [2, 120, 121]. When examining the effect of the breadth of the autoantibody profile, early RA patients who were positive for ACPA, anti‐CarP antibodies, as well as AAPA showed more radiographic progression than patients who were positive for only one AMPA at 12 months follow‐up [122]. No significant differences in radiographic progression were found between patients with one AMPA and those who were AMPA negative [122]. In another study, similar associations with radiographic progression were observed for triple‐positive patients compared to those positive for only one AMPA [121]. However, analysis of whether this association was driven by the number of AMPA or a specific AMPA revealed that there were no differences in radiographic progression between ACPA‐positive patients with and without additional AMPA, indicating that radiographic progression is mainly associated with ACPA positivity [121]. Overall, the presence of ACPA appears to be the most important biomarker for radiographic progression in RA patients, while measuring the other AMPA seems to provide limited additional information.

Moreover, ACPA‐positive patients are less likely to achieve DFR compared to ACPA‐negative patients [110, 111]. In addition, a broader autoantibody profile at baseline, reflected by the number of ACPA, anti‐CarP, AAPA, and RF isotypes, was also associated with reduced rates of DFR [112]. In this study, no associations were found for any specific AMPA isotype. In contrast, another study reported that ACPA IgA was associated with a lower rate of DFR, but that this risk could be explained by the concurrent presence of ACPA IgG [123]. Consistent with findings on DFR, ACPA‐positive patients achieve less frequently SDFR than ACPA‐negative patients (5%–10% vs. 40%, respectively) [124]. In contrast, the breadth of the autoantibody profile was not associated with SDFR [112, 121].

In conclusion, when it comes to drug‐free remission, be it sustained or not, ACPA is a key negative predictor, meaning that ACPA‐positive patients are less likely to achieve this outcome. Intriguingly, a broader autoantibody profile, despite being associated with a better early treatment response, is associated with a smaller chance of DFR. This may indicate that the inflammation caused by an active B cell/plasma cell response (which is the presumed underlying equivalent of a broad autoantibody response) is amenable to early immunosuppression, but that it is ultimately not entirely quenchable.

### 6.4 Changes in AMPA Levels During the Disease Course

Various studies have examined whether changes in AMPA levels are associated with disease activity and long‐term outcomes. A decrease of AMPA levels during the first year of treatment has been consistently reported [118, 125, 126]. These changes were not associated with disease activity or long‐term clinical outcomes, but instead were linked to the intensity of immunosuppressive treatment [118, 126]. Seroconversion from ACPA‐positive to ACPA‐negative was rarely observed in RA patients and was not associated with clinical outcomes [127, 128]. Altogether, these findings indicate that once AMPA are present, changes in AMPA levels do not reflect ongoing disease activity. The initial decrease in autoantibody levels could be attributed to effects of immunosuppressive treatment, while the persistent presence of AMPA may be partly driven by autoantibody production by long‐lived plasma cells in the bone marrow, which are not strongly affected by immunosuppressive treatment.

In summary, the clinical presentation of seropositive and seronegative RA is highly similar, with some differences observed for the presence of anti‐CarP antibodies and the breadth of the autoantibody profile. Treatment responses are also largely comparable between the two groups, although seropositive patients with a broader autoantibody profile may show a better early treatment response. In contrast, clear differences emerge in the long‐term, with seropositive RA being associated with poorer clinical outcomes. Once AMPA are present, longitudinal analysis of AMPA levels in patients with established RA appears to provide limited added value for predicting long‐term outcomes. From a pathophysiological perspective, these findings suggest that seropositive and seronegative disease may be partly driven by distinct underlying immune mechanisms. For clinical practice, these findings indicate that ACPA positivity is a relevant biomarker for disease severity, but not necessarily for predicting treatment response. Therefore, ACPA are useful for distinguishing clinical phenotypes, but may have limited value in guiding treatment decisions. Overall, AMPA represent interesting biomarkers in RA, as they provide insights into disease heterogeneity and underlying disease mechanisms.

## 7 Pathogenic and Protective AMPA

**FIGURE 3:**
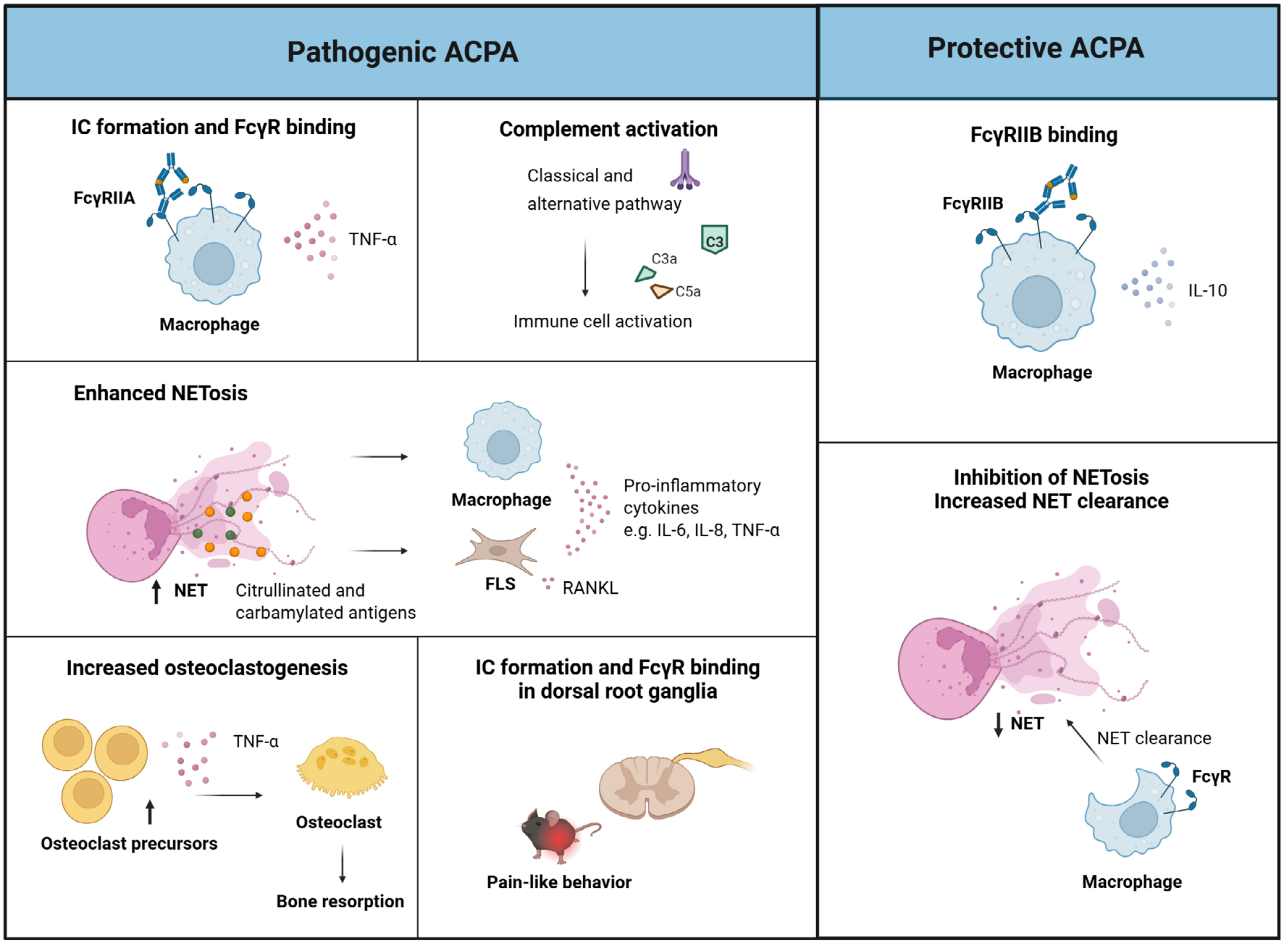
Pathogenic and protective effects of ACPA. Schematic overview of proposed mechanisms of pathogenic and protective effects of ACPA. Left: Mechanisms through which pathogenic, pro‐inflammatory effects may be regulated include immune complex (IC) formation and binding to FcγRs on macrophages, complement activation, and enhanced NETosis. Neutrophil extracellular traps (NETs) are a potential source for citrullinated and carbamylated autoantigens and can activate macrophages and fibroblast‐like synoviocytes (FLS) to produce pro‐inflammatory cytokines. ACPA can promote osteoclast differentiation, resulting in increased bone resorption and subsequent bone loss. ACPA‐induced pain‐like behavior in mice is potentially mediated by IC formation and FcγR binding on macrophages in dorsal root ganglia. Right: Protective effects of ACPA are regulated via IC formation and binding to FcγRIIB on macrophages, inhibition of NET release, and enhanced NET clearance. Additional studies are needed to clarify the illustrated mechanisms. Created in BioRender. Stalman (2026). https://BioRender.com/4k8m12y.

Given the strong associations of AMPA with both risk factors and disease progression, they have been widely hypothesized to be pathogenic in RA. Several mechanisms underlying their role in RA pathogenesis have been proposed, with most mechanistic insights derived from studies on ACPA. More recently, studies have suggested that ACPA may also exert protective functions. This section outlines current insights into potential mechanisms underlying the pathogenic and protective roles of AMPA in RA (Figure 3).

### 7.1 Fc Gamma Receptor Binding and Complement Activation

Important antibody effector functions include binding to Fc gamma receptors (FcγRs) expressed on immune cells to induce cellular responses, and activation of the complement system. ACPA have been shown to form immune complexes with citrullinated fibrinogen. These ACPA immune complexes activated macrophages to secrete TNF‐α, a key pro‐inflammatory marker in RA [129, 130, 131]. This was dependent on the interaction with FcγRIIA and TLR‐4 on macrophages, with this latter finding raising the question to which extent these immune complexes contained LPS [129, 130, 131]. In the presence of RF IgM or IgA, this pro‐inflammatory response was found to be enhanced [132]. Furthermore, ACPA immune complexes were shown to bind FcγRI expressed on activated neutrophils [133]. Given that neutrophils are abundantly present in the synovial fluid of RA patients, this interaction may play a role in RA pathogenesis [133].

Moreover, increased levels of complement products can be detected in plasma and synovial fluid of RA patients [134, 135]. In line with this, ACPA in serum of RA patients has been demonstrated to initiate the complement system through both the classical and alternative pathway, but not the lectin pathway [136]. Similar to activation via FcγR binding, activation of the complement system was enhanced in the presence of RF [132].

### 7.2 Neutrophil Extracellular Traps

Another mechanism implicated in RA pathogenesis is NETosis. NETosis is the formation and release of neutrophil extracellular traps (NETs), which are extracellular structures composed of decondensed chromatin and neutrophil‐derived antimicrobial proteins. Increased NETosis has been observed in neutrophils derived from blood and synovial fluid of RA patients [137, 138]. This increased NETosis correlated with ACPA levels and systemic inflammation markers, including CRP, ESR, and IL‐17 [137]. In vitro induced NETosis resulted in the release of citrullinated autoantigens, such as vimentin and α‐enolase, which have been shown to be targeted by ACPA of RA patients [137]. In addition, B cells that were differentiated in synovial ectopic lymphoid structures showed reactivity toward citrullinated antigens that are potentially released by NETosis [139]. Moreover, ACPA could further enhance NETosis in neutrophils [137]. In turn, NETs activated fibroblast‐like synoviocytes, inducing the release of pro‐inflammatory cytokines and chemokines, including IL‐6, IL‐8, CCL20, and ICAM‐1. Taken together, these findings suggest that NETs are a potential source of citrullinated autoantigens that can drive the ACPA response, while NETs can also enhance pro‐inflammatory immune responses in the joints [137].

Furthermore, immunofluorescence detection revealed that carbamylated proteins can also be present in NETs [140]. Proteomic analysis identified the presence of carbamylated histones. Anti‐CarP antibodies directed against carbamylated histones, vimentin, and α‐enolase could be detected in RA patients. Additionally, upon immunization of mice with carbamylated NETs, anti‐CarP antibodies could be induced in vivo. Another study identified antibodies against carbamylated LL37, which is another antimicrobial peptide externalized during NETosis [141]. Thus, NETs may also be a source of carbamylated autoantigens that can potentially drive anti‐CarP responses [140]. Interestingly, levels of anti‐CarP antibodies against carbamylated histones and LL37 correlated with bone erosion scores in RA patients [140, 141]. Incubation of carbamylated NETs with macrophages resulted in the release of pro‐inflammatory cytokines, including IL‐6, IL‐8, and TNF, while incubation with fibroblast‐like synoviocytes (FLS) promoted osteoclast formation via the release of RANKL [140]. Additionally, anti‐CarP immune complexes enhanced osteoclast formation and activation [140, 141]. Thus, these findings suggest a potential role for NETs in the mechanism underlying the association between anti‐CarP antibodies and bone erosion.

### 7.3 Bone Loss and Pain

ACPA have been strongly associated with joint destruction in RA patients [4]. Although joint destruction is often considered a consequence of chronic inflammation, ACPA may also contribute to bone loss by activating osteoclasts and increasing bone resorption [142, 143]. In ACPA‐positive patients, levels of bone resorption markers were increased compared to ACPA‐negative patients and controls, while levels of bone formation markers remained similar [142]. ACPA with reactivity to vimentin were shown to bind osteoclasts and promote osteoclastogenesis and bone resorption in vitro [142]. Additionally, adoptive transfer of human ACPA into mice promoted bone loss through enhanced osteoclastogenesis and bone resorption [142]. This effect was mediated through the release of TNF‐α by osteoclast precursors, a cytokine that enhances osteoclastogenesis. Moreover, another study showed that polyclonal ACPA isolated from peripheral blood or synovial fluid of RA patients induced differentiation of macrophages into osteoclasts in vitro [144]. Findings in this study suggested that the effect of ACPA on osteoclastogenesis is mediated by IL‐8. However, it appeared that some of the tested monoclonal ACPA lacked specificity for citrullinated peptides; hence, additional studies are needed to elucidate these findings [145].

Moreover, studies have investigated whether ACPA may be linked to pain induction in RA. Polyclonal human ACPA were shown to induce pain‐like behavior in mice [146]. Notably, these mice showed no signs of joint inflammation. Furthermore, injection of monoclonal ACPA also resulted in pain‐like behavior in mice via activation of osteoclasts and subsequent IL‐8 release [146]. However, as mentioned previously, some of these monoclonal antibodies lacked specificity, indicating that the observed effects could not be conclusively attributed to ACPA. A subsequent study demonstrated that the injection of two monoclonal ACPA derived from RA patients (C03 and B09) induced pain‐like behavior in mice [147]. However, injection of control antibodies could also induce pain‐like behavior, although this was transient and less pronounced. This suggests that the observed effects may not be entirely ACPA‐specific. Additionally, trabecular bone loss and subclinical tenosynovitis were also observed in these mice. These effects were diminished in PAD4‐deficient mice, suggesting that the underlying mechanisms of pain‐like behavior, bone loss, and inflammation in this model are dependent on citrullination by PAD4. Further investigations revealed that the induced pain‐like behavior observed in mice after B09 injection may depend on FcγR activation [148]. B09 was found to accumulate in the dorsal root ganglia in mice, where it colocalized with satellite glia cells. In addition, B09 increased the expression of genes related to satellite glia cells, neurons, and macrophages. Based on these findings, it was suggested that B09 may induce pain‐like behavior in mice by forming immune complexes and activating macrophages through FcγR binding in the dorsal root ganglia. In light of these in vivo findings, studies have also investigated whether an association exists between ACPA and pain in patients with early and established RA [149, 150]. In these studies, however, no differences in pain were observed between ACPA‐positive and ACPA‐negative RA patients. Thus, although in vivo studies have established a link between ACPA and pain‐like behavior in mice, the clinical relevance of this association for clinical practice remains to be clarified.

### 7.4 Protective ACPA

Recent studies have suggested that ACPA may also exert protective effects in RA. For instance, an engineered therapeutic ACPA targeting citrullinated histones H2A and H4 showed anti‐inflammatory effects in multiple mouse models, including arthritis models [151]. These effects were mediated through inhibition of NET formation and enhanced Fc‐mediated clearance of NETs and neutrophils by macrophages [152].

Intriguing findings were reported by recent studies examining the in vivo effects of monoclonal ACPA, which were generated from ACPA BCR sequences derived from patients with established RA. In contrast to what might have been expected based on the in vitro experiments described above, intravenous administration of monoclonal ACPA did not induce arthritis or pain‐like behavior in mice [153]. Instead, ACPA clone E4 protected against antibody‐induced arthritis. This protective effect was restricted to inflammation in joints, and was shown to be mediated through FcγRIIB binding on macrophages. Treatment with E4 resulted in a reduced number of osteoclasts in vivo and inhibited osteoclast differentiation in vitro. Furthermore, E4 could form immune complexes with citrullinated α‐enolase, and these immune complexes induced increased secretion of IL‐10 by murine or human‐derived macrophages in vitro.

Consistent with these findings, two other studies reported amelioration of arthritis following administration of monoclonal ACPA in vivo [154, 155]. Both studies used collagen antibody‐induced arthritis (CAIA) mouse models. In one study, several monoclonal ACPA clones inhibited or reduced arthritis, whereas others had no effect and one increased disease severity [154]. Additionally, therapeutic administration of one ACPA clone almost completely resolved arthritis. The anti‐inflammatory effect was dependent on Fc‐FcγR interactions, since an Fc region capable of binding FcγRs was required for the anti‐inflammatory effect to occur. Additionally, administration of polyclonal ACPA derived from RA patients resulted in overall enhanced anti‐inflammatory effects compared to a control pool without ACPA. Similarly, the second study showed that administration of ACPA prevented development of arthritis and reduced disease severity when administered at an early disease stage [155]. However, this effect was not observed when ACPA were administered at later stages, indicating that the protective effect was confined to early disease stages in this mouse model.

In summary, several pathways via which ACPA could exert pathogenic, pro‐inflammatory functions have been described. ACPA can form immune complexes and bind to FcγRs on immune cells. Additionally, ACPA can activate the complement system and increase NETosis. NETs were shown to be a potential source for citrullinated and carbamylated autoantigens. Furthermore, ACPA have been linked to bone loss and are suggested to play a role in pain induction. On the other hand, recent insights suggest that some ACPA may have protective functions. Two potential mechanisms have been proposed. One involves the inhibition of NET release together with enhanced NET clearance through immune complex formation and FcγR binding on macrophages. The other also involves immune complex formation and binding to FcγRIIB on macrophages, thereby promoting IL‐10 production.

It remains to be elucidated how some ACPA can have pro‐inflammatory effects, whereas others display anti‐inflammatory effects. A feature that is hypothesized to explain these different functions is ACPA specificity, particularly the distinction between promiscuous and private ACPA [9]. Promiscuous ACPA recognize multiple citrullinated epitopes, whereas private ACPA display a more restricted specificity. Current studies have not (yet) established a pathogenic role for promiscuous ACPA. Instead, some promiscuous ACPA have been reported to exert protective effects. In contrast, private ACPA that recognize citrullinated epitopes in the joints or are cross‐reactive with joint proteins may potentially be pathogenic [9]. In addition to studies on the functional roles of ACPA, it may also be of interest to investigate other AMPA, such as anti‐CarP antibodies and AAPA. However, the broad cross‐reactivity of AMPA may complicate the distinction between mechanisms that are specific or shared across different AMPA. Finally, it would be valuable to gain insights into the disease stages at which pathogenic and protective effects of AMPA may be relevant, for example whether protective ACPA are involved in ACPA‐positive healthy individuals or influence disease progression once RA is established [156].

## 8 Conclusion

AMPA are associated with disease development and progression. Genetic and environmental risk factors are mainly associated with seropositive disease and act at different stages of disease development. Maturation of the AMPA response is observed before the onset of symptoms and is hypothesized to drive progression to disease. Furthermore, although the initial clinical presentation and treatment response are largely similar between seropositive and seronegative RA, seropositive RA is associated with poorer long‐term clinical outcomes. Together, these findings indicate that AMPA are relevant biomarkers in RA and may reflect underlying disease processes. While research has primarily focused on the pathogenic effects of ACPA, recent studies have shown that ACPA can also have protective effects. This suggests that AMPA may have diverse functions: some promote inflammation, others contribute to anti‐inflammatory responses, while others reflect ongoing immune responses without evident functional effects. However, whether AMPA directly contribute to RA pathogenesis still remains unclear. Further studies are needed to elucidate the pathogenic and protective functions of AMPA and the factors underlying these functional differences. Ultimately, these insights will contribute to our understanding of the role of AMPA in RA.

## Funding

This work was supported by a Vidi grant from the NWO (Dutch Research Council, 09150172110053 to DvdW) and Horizon Europe (Project ID: 101136582).

## Conflicts of Interest

The authors declare no conflicts of interest.

## Data Availability

Data sharing not applicable to this article as no datasets were generated or analyzed during the current study.
